# CD11b^+^ cells markedly express the itch cytokine interleukin‐31 in polymorphic light eruption

**DOI:** 10.1111/bjd.18092

**Published:** 2019-07-21

**Authors:** V. Patra, J. Strobl, A. Gruber‐Wackernagel, P. Vieyra‐Garcia, G. Stary, P. Wolf

**Affiliations:** ^1^ Research Unit for Photodermatology Medical University of Graz Graz Austria; ^2^ Center for Medical Research Medical University of Graz Graz Austria; ^3^ Department of Dermatology Medical University of Vienna Vienna Austria; ^4^ CeMM Research Center for Molecular Medicine of the Austrian Academy of Sciences Vienna Austria; ^5^ Ludwig Boltzmann Institute for Rare and Undiagnosed Diseases

Dear Editor, Itch is one of the cardinal symptoms of polymorphic light eruption (PLE), the most common form of photodermatosis known to be mediated immunologically.^1^, ^2^, ^3^ Indeed, itch often precedes the skin lesions or may even be the only symptom in PLE,^4^ and is sometimes aggravated to a burning sensation. There have been reports of a variant called PLE sine eruption, with intense pruritus on sun‐exposed areas without any visible skin changes.^4^ However, the underlying cause and cellular mechanisms of itch in PLE are not known. Interleukin (IL)‐31 is a novel cytokine of the IL‐6 family, also described as a ‘pruritogenic cytokine’ owing to its link between the immune and neuronal systems to induce itch.^5^ IL‐31 is expressed by a variety of inflammatory cells.^5^ It binds to the IL‐31 receptor alpha complex (IL‐31RA), and mediates inflammatory itch by forming a functional receptor through coupling to oncostatin M receptor (OSMR)β.^6^

We examined IL‐31, IL‐31RA and OSMR expression by immunohistochemistry and immunofluorescence on archived formalin‐fixed, paraffin embedded samples obtained from our tissue bank, which were reported in a previous study.^7^ The samples comprised lesional skin of 12 women and one man (age range 16–76 years) with photoprovoked PLE (eight had undergone UVA testing, three UVB testing, one UVB phototherapy and one natural sunlight exposure, with PLE occurring in all within 1–3 days after exposure). In addition we analysed samples from eight people (one woman, seven men; age range 6–63 years) with subacute to chronic atopic dermatitis (AD) and eight (seven women, one man; age range 31–74) years with chronic plaque psoriasis. Healthy‐appearing skin samples from tumour‐adjacent sites obtained by surgical excision of lesions such as naevi and nonmelanoma skin cancers of 10 patients (five women, five men; age range 51–87 years) were used as control. The investigations were in accordance with protocols approved by the Ethics Committee of Medical University of Graz, Graz, Austria (18‐068 ex 06/07 and 25‐293 ex 12/13) and the guidelines of the Declaration of Helsinki Principles.

**Figure 1:**
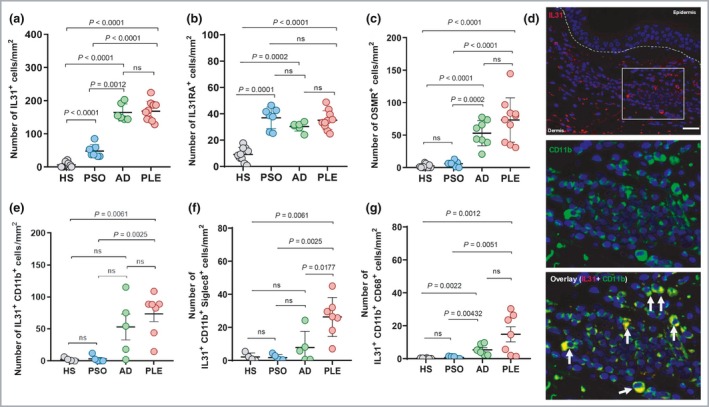
The majority of interleukin (IL)‐31 expression in polymorphic light eruption (PLE) is by CD11b^+^ cells. Quantitative analysis of immunohistochemical staining showing the number of (a) interleukin (IL)31^+^ cells; (b) IL‐31 receptor alpha complex (IL31RA)^+^ cells; and (c) oncostatin M receptor (OSMR)^+^ cells in different diseases. Visual analysis (a–c) was performed by counting positively stained cells in five of the most densely infiltrated microscopic fields randomly selected from the same section, at a magnification of × 40. (d) Representative images of double staining showing high numbers of CD11b^+^ cells expressing IL31 in PLE. (e–g) Quantitative analysis showing the number of (e) IL‐31‐expressing CD11b^+^, (f) IL31 expressing CD11b^+^ Siglec8^+^ (eosinophils); and (g) CD11b^+^ CD68^+^ cells (macrophages). Automated analysis for immunofluorescence (e–g) was performed using TissueQuest image analysis software v6·0 (TissueGnostics GmbH, Vienna, Austria). Briefly, nuclei were assessed by 4,6‐diamidino‐2‐phenylindole mean intensity and area measurement, while cell surface and intracellular markers were detected inside and around nuclear staining, respectively. Number of positive cells/mm² was calculated and used for statistical analysis. Data are presented as mean ± SD. *P* values were determined by Mann–Whitney test. (f) All *P* values except one [atopic dermatitis (AD) vs PLE] remained significant after Bonferroni correction, setting significance to *P* ≤ 0·0083; ns, not significant. Scale bar, 50 μm.

Following heat‐induced antigen retrieval, staining was performed with peroxidise/3‐amino‐9‐ethylcarbazol (AEC) (REAL™ Detection system; Dako, Glostrup, Denmark), using antibodies directed against IL‐31 (1 : 200, #GTX85642; GeneTex, Irvine, CA, USA), IL‐31RA (1 : 200, #ab113498; Abcam, Cambridge UK), and OSMR (1 : 20, #10982‐1‐AP, Proteintech, Rosemont, IL, USA). Expression levels (mean ± SD) of IL‐31, IL‐31 RA and OSMR in lesional skin of PLE (168·0 ± 29·8, 35·1 ± 7·2 and 73·2 ± 33·9 cells/mm^2^, respectively) were similar to those in AD (164·4 ± 26·0, 30·3 ± 3·4 and 52·9 ± 19·5 cells/mm^2^) and higher than in healthy skin (6·3 ± 7·4, 9·1 ± 5·0 and 2·4 ± 2·8 cells/mm^2^) (Fig. 1a–c). In psoriatic skin, overall expression of IL‐31 (48·0 ± 19·4 cells/mm^2^) and OSMR (5·9 ± 3·9 cells/mm^2^) was lower compared with PLE, while IL‐31RA (36·9 ± 8·2 cells/mm^2^) was expressed at similar levels. High numbers of IL‐31^+^, IL‐31RA^+^ and OSMR^+^ cells were observed in polymorphonuclear leucocyte infiltrations in the dermis and in blood vessels (mostly in PLE lesions) (data not shown). There was no expression of IL‐31 in the epidermis (Fig 1d).

To identify the cellular sources of IL‐31 in the different disease conditions, we performed multicolour staining using conjugated monoclonal antibodies against IL‐31 (1:30, clone: 1D10B31, #659603), CD68 (1:20, clone: Y1/82A, #333810), Siglec8 (1:20, clone: 7C9, #347106) (all Biolegend, San Diego, CA, USA), CD11b (1:20, clone: Bear1, #IM0530; Beckmann Coulter, Brea, CA, SA), and 4,6‐diamidino‐2‐phenylindole nuclear marker (Roche Applied Sciences; Indianapolis, IN, USA). Double staining indicated that the major source of IL‐31 in PLE was mainly CD11b^+^ cells (73·8± 32·5 cells/mm^2^) (Fig. 1d, e). Triple staining indicated that a substantial portion of IL‐31^+^ CD11b^+^ cells were also positive for Siglec8 (eosinophilic marker) (Fig. 1f) or CD68 (macrophage marker) (Fig. 1g). The numbers of those cells were significantly higher in PLE lesions compared with healthy or psoriatic skin (Fig. 1e–g).

A previous study showed CD11b^+^ cells in skin of people with PLE and enhanced infiltration of these cells upon UV exposure in lesional skin, and found that most of these CD11b^+^ cells were CD68^+^ macrophage‐like cells.^1^ Furthermore, PLE lesions are sometimes infiltrated with eosinophils.^4^ In our study, we observed elevated numbers of macrophages and eosinophils expressing IL‐31 in PLE lesions (Fig. 1f, g), in levels nearly similar to AD.^6^

IL‐31 is known to be induced by exposure to UV radiation, and its potential mediators including human beta‐defensins (HBDs) and LL‐37.^6^ We have previously reported increased HBD‐2 and LL‐37 in PLE lesions.^7^ Certain antimicrobial peptides can augment the production of IL‐31 through a positive loop response and thus could contribute to the development of the itchy lesions in PLE.^6^ Interestingly, macrophages that were stimulated by microbial elements such as staphylococcal exotoxins [staphylococcal enterotoxin B (SEB), alpha‐toxin] were able to significantly upregulate IL‐31RA.^6^ Microbial elements are hypothesized to be involved in the pathogenesis of PLE.^8^ Furthermore, macrophages and eosinophils treated with SEB and IL‐31 can secrete pro‐inflammatory cytokines such as IL‐1β.^6^ In this regard, Lembo *et al*. have shown increased expression of IL‐1 family members in PLE.^2^

Although this study has limitations such as overall small sample size and imperfect age and sex matching, its findings may open new avenues for the development of novel treatment strategies in PLE, targeting IL‐31. Indeed, anti‐IL‐31 blockade has been designed for treating itch and the monoclonal anti‐IL‐31 receptor antibody nemolizumab has been successfully used to neutralize the itch in patients with moderate‐to‐severe atopic dermatitis.^6^
